# Serotype Distribution and Antimicrobial Sensitivity Profile of *Streptococcus pneumoniae* Carried in Healthy Toddlers before PCV13 Introduction in Niamey, Niger

**DOI:** 10.1371/journal.pone.0169547

**Published:** 2017-01-19

**Authors:** Sani Ousmane, Bouli A. Diallo, Rasmata Ouedraogo, Abdel-Kader A. Sanda, Amadou M. Soussou, Jean-Marc Collard

**Affiliations:** 1 Centre de Recherche Médicale et Sanitaire, CERMES, Niamey, Niger; 2 Université Abdou Moumouni, Faculté des Sciences et Technique, Niamey, Niger; 3 Centre Hospitalier Universitaire Pédiatrique Charles-de-Gaulle, Ouagadougou, Unité de Formation et de Recherche en Sciences de la Santé, Ouagadougou, Burkina Faso; Azienda Ospedaliera Universitaria di Perugia, ITALY

## Abstract

**Background:**

To mitigate the burden of pneumococcal infections in Niger, a 13-valent pneumococcal vaccine, PCV13, was introduced for routine child vaccination in July 2014. In order to provide pre-vaccine baseline data and allow appreciation of changes on carriage due to vaccination, we analyzed retrospectively pneumococcal isolates obtained from healthy, 0 to 2 year old children prior to the vaccine introduction.

**Methods:**

From June 5, 2007, to May 26, 2008, 1200 nasopharyngeal swabs were collected from healthy 0 to 2 year old children and analyzed by standard microbiological methods. Serotyping was done by SM-PCR and the data were analyzed with R version 2.15.0 (2012-03-30).

**Results:**

*Streptococcus pneumoniae* was detected in 654/1200 children (54.5%) among whom 339 (51.8%) were males. The ages of the study subjects varied from few days to 26 months (mean = 7.1, median = 6, 95% CI [6.8–7.4]). Out of 654 frozen isolates, 377 (54.8%) were able to be re-grown and analyzed. In total, 32 different serogroups/serotypes were detected of which, the most prevalent were 6/(6A/6B/6C/6D) (15.6%), 23F (10.6%), 19F (9.3%), 14 (9%), 19A (5.6%), 23B (4.0%), 25F/38 (3.7%), 18/(18A/18B/18C/18F) (2.9%) and PCR non-typeable (16.4%). Eleven serogroups/serotypes accounting for 57.3% (216/377) were of PCV13 types. Of the 211/377 (56%) isolates tested for drug sensitivity, 23/211 (10.9%), 24/211 (11.4%), 9/211(4.3%) and 148/210 (70.5%) were respectively resistance to oxacillin, chloramphenicol, erythromycin and tetracycline. Thirteen of the oxacillin resistant isolates were additionally multidrug-resistant. No resistance was however detected to gentamycin_**500μg**_ and to fluoroquinolones (ø Norfloxacin_**5μg**_ <7mm). Age > 3 months and presence in family of more than one sibling aged < 6 years were significant risk factors for carriage.

**Conclusion:**

A global rate of 54.5% pneumococcal carriage was detected in this study. The introduced PCV13 vaccine should cover 57.3% (216/377) of circulating serogroups/serotypes, among which were those resistant to antibiotics. Age > 3 months and presence in family of children aged < 6 years were significant factors for pneumococcal carriage. The present data should help understanding post vaccine introduction changes in pneumococcal carriage and infections for better action.

## Introduction

*Streptococcus pneumoniae* (SPN) is a human commensal and pathogen that is asymptomatically carried in the nasopharynx from as early as the first month of life [1]. Although carriage is unstable and mostly self-resolving, it constitutes a prerequisite to invasive infections by SPN [2]. Despite host defense, SPN can opportunistically invade nearby or distant sites of the host including the ear, the lower respiratory tract, blood and cerebrospinal fluid (CSF), and cause disease such as otitis media, bacteremia, septicemia and meningitis [3]. SPN avoids host defense and invades tissues by use of many mechanisms and virulence factors among which is capsular polysaccharide that surrounds the cell wall and which is differentiated into more than 91 antigenic types [4]. In immunocompetent individuals, capsular polysaccharide induces antibody production but not memory cell response. Pneumococcal polysaccharides have been used as vaccines as early as 1970 [5]. But, to make it potentially protective in < 2 year old children, the capsular component is linked to a carrier protein (conjugation) that can induce T-cell immunity and provide longer protection even in toddlers [6].

Despite being vaccine-preventable, pneumococcal infections continue to impose a serious health challenge to low and middle-income countries due to limited access to and coverage of vaccines. It has been estimated that from 2009 to 2011 the proportion of confirmed meningitis due to SPN in the African meningitis belt rose from 12.8% to 47.6% with potential to cause epidemics [7, 8]. To combat pneumococcal infections, many developing countries are introducing conjugate vaccines in their routine immunization programs (EPI) [7]. The success of pneumococcal conjugate vaccines (PCVs) in preventing vaccine serotype (VT) disease is largely due to their efficacy to prevent carriage in both vaccinated and their contacts. For that reason, carriage surveys, mostly considered relatively cheaper compared to population-based surveillance in low and middle-income countries, are conducted to indirectly monitor vaccination effects, herd immunity, serotype replacement and resistance to antibiotics [8–11]

In Niger, PCV13 was introduced in July 2014 for the routine immunization of 0 to 2 year old children. Prior to this introduction and to the limit of our research, there were no available data on the epidemiology of pneumococcal carriage, particularly among the PCV13 vaccine target group of children. However, in a previous study on pneumococcal meningitis conducted by Collard et al. [7] in 2013 before PCV13 introduction in Niger, the authors predicted 68.7% vaccine coverage rate and showed that serotype1, a serotype seldom isolated in carriage, was the predominant cause (43.5%) of meningitis.

The aim of the present study was to provide pre PCV13 start baseline data on pneumococcal carriage in toddlers and allow appreciation of changes in epidemiology of that carriage and infections as a result of the vaccine introduction.

## Materials and Methods

### Study Site

The study subjects were enrolled in the maternal and infant protection health center, PMI Yantala Haut, a public, primary health care center delivering care services for all but mainly for mothers and children. In 2007, the center covered a population of about 53.224 inhabitants in a catchment area of 0–5 km within commune I of Niamey, the capital city of Niger. PMI Yantala is a close neighbor of *Centre de Recherche Médicale et Sanitaire*, CERMES, a public but autonomous health research institute where the this study was conducted. CERMES was created to carry out research on meningitis and schistosomiasis since 1980 and had undergone important structural and administrative changes in 2000 to include activities in public health, research and training of health personnel. The center had been first an associate member of International Network of Pasteur Institutes (RIIP) from 2003 to 2007 before becoming a full member of the network in 2007.

### Participants and Sample Collection

The study was conducted from June 5, 2007, to May 26, 2008 and enrolled healthy children taken to PMI Yantala Haut health center for routine systematic vaccination. The criteria for a child to be enrolled were being aged between 0 to 2 year old, being healthy without any history of infectious disease in the last 3 months to 7 days before the enrollment date and obtaining signed consent of parents, next of kin or of a person having witnessed the parent’s or next of kin’s oral consent. A questionnaire form was then addressed to mothers or next of kin for the collection of socio-demographic information. Nasopharyngeal samples were subsequently collected once for each participant according to WHO recommendations [12] by the study physician using a flexible cotton swab (Calgiswab, COPAN Strasbourg). The inclusion was done once in a given day of each week, throughout the study period. The study protocol, the consent and questionnaire forms were all submitted to and approved by the Niger National Consultative Committee of Ethic through deliberation N° 02/2007/CCNE of February1, 2007.

### Microbiological Analysis

Collected swabs were immediately inoculated on plates of Colombia agar medium containing 15 mg/l Nalidixic acid (Bio-Rad Ref. 60534) and supplemented with 7–10% sheep blood. Plates were then incubated at 36±0.5°C under approximate 5% CO2 atmosphere (GENbag BioMérieux®. Ref. 45533) for18 ± 2 hours. Suspected colonies were further selected and purified on new blood agar plates on which an optochin disc (OPT-F BioMérieux®. Ref.55912) was applied and incubated under the same conditions as stated above. SPN were identified as a Gram-positive diplococci, α-hemolytic, oxydase and catalase negative colonies with an optochin inhibition zone ≥14 mm (Ref. SFM-CA edition 2008) and further confirmed by latex agglutination using Slidex® Pneumo-Kit (BioMérieux Ref. 58821). All confirmed SPN strains were kept at -80°C in Trypto-Casein-Soya (TCS) broth containing 0.5% glucose, 2% skimmed milk and 10% glycerol until use. Serotyping was performed by Sequential Multiplex PCR (SM-PCR) as described by Collard et al [7] with little change in that samples consist of pneumococcal isolates rather than CSF and PCR run in 12 reactions rather than 8. DNA extracts were obtained by suspending frozen isolates in 500μl PBS buffer and heating to 100°C for 10 minutes. Antimicrobial sensitivity testing was performed using the Kirby–Bauer disc diffusion method, based on recommendations of the French Committee for Antibiotic sensitivity testing (CA-SFM, 2013). Inhibition zones were read with OSIRIS (Expert version 3.2, BIO-RAD) with manual adjustment where necessary. Minimum Inhibitory Concentrations (MIC) were measured using BioMérieux E-tests (BioMérieux). In this study, an isolate was considered less susceptible to penicillin when an inhibition zone of less than 26 mm was observed around oxacillin_**5μg**_ disc and resistant when in addition the measured MIC of cefotaxim was > 0.5μg (CA-SFM, 2013 edition).

### Data Analysis

All data collected have been confidentially recorded into a MySQL database and extracted to Excel for analysis with open source R v.2.15.0 (2012-03-30); The R Foundation for Statistical Computing, ISBN 3-900051-07 0. X^2^ test was used to compare frequencies or means. Risk factors for SPN carriage were first analyzed individually by univariate (single variable) analysis then examined together in multivariable (multiple variables) analysis by logistic regression model.

### Ethics Statement

The study was conducted in accordance with the then last Helsinki Declaration and started after approval obtained from the Niger National Consultative Committee of Ethic through deliberation N° 02/2007/CCNE of February1, 2007. Detailed information about the study aim, the sampling method and its related risk, the use and confidentiality of data were all made clear to parents or to next of kin before obtaining and signing their consent. Parents or next of kin were free to refuse the participation of their child, to ask question or to have access to the laboratory result when they wished. Sample collection, analysis and waste management were carried out according to WHO recommendations[12]

## Results

### Characteristics of the Participants

Overall, 1200 healthy children have been enrolled of whom 629 (52.4%) were males. Age was known for 1193/1200 (99.4%) and varied from 0 month (few days) to 26 months with mean age = 7.1 months, median = 6 months (95% CI [6.9–7.5]). The lowest and maximum birth weight recorded were respectively 1320g and 4350g, mean = 2999g. Of the 1200 enrolled children, 430 (35.8%) had no sibling, 767 (63.9%) lived with at least one and 3 (0.3%) had unknown sibling status. Eighty-nine (7.4%) and 1093 (91.1%) were breastfed for less than and for at least 2 months respectively. We also recorded 469 children (39.1%) that were living in a smoking environment. Children with a history of infection at least 7 days before the enrollment were 562 (46.8%). Those that received antibiotic treatment 7 days to the inclusion date were 155 (12.9%). Mothers of the study subjects comprised of 17 ethnic groups with Zarma-Sonrai, Hausa, Tuareg, Burkinabese and Beninese respectively representing 49.1%, 25.8%, 7.9%, 3%, and 2.1%.

### Pneumococcal Carriage

**Fig 1:**
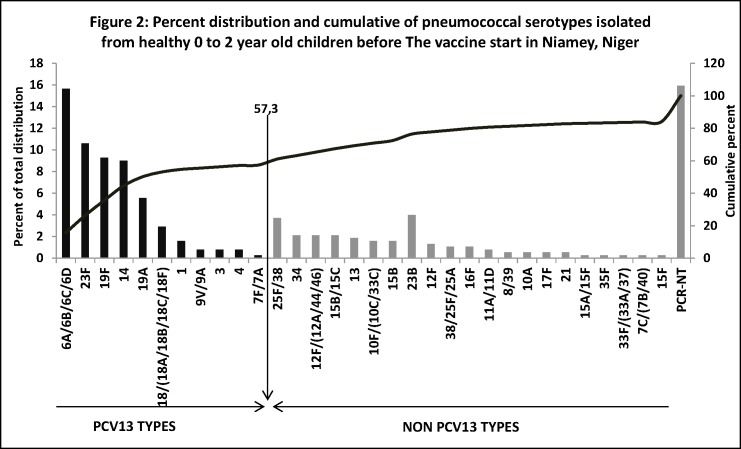
Percent distribution (Bar charts) and cumulative (curve) of pneumococcal carriage serotypes isolated from healthy, 0 to 2 year old children before PCV13 introduction in Niamey Niger. Legend: Dark bars represent serogroups/serotypes covered by PCV13 vaccine; gray bars represent non PCV13 vaccine serogroups/serotypes; curve represents cumulative percentage of serogroups/serotypes distribution.

Globally, SPN was carried by 654/1200 children (54.5%) among whom 339 (51.8%) were males. Of the 654 frozen isolates, only 377 (57.6%) were able to be re- grown for antimicrobial sensitivity testing and molecular typing. In total, 32 serogroups/serotypes were identified and the most prevalent were serogroups/serotypes 6/(6A/6B/6D/6C), 23F, 19F, 14, 19A, 23B, 25F/38,18/(18A/18B/18C/18F) and PCR-non-typeable (PCR-NT), respectively representing 15.6%, 10.6%, 9.3%, 9%, 5.6%, 4%, 3,7%, 2.9 and 16.4% (Table 1). Eleven serogroups/serotypes cumulating to 216/377 isolates (57.3%) were of PCV13 vaccine-types (Fig 1).

**Table 1:** Number and percent distribution of pneumococcal carriage serogroups/serotypes isolated from healthy 0 to 2 year old children before PCV13 start in Niamey, Niger.

Serogroup/serotype	Number	Percent
6/(6A/6B/6C/6D)	59	15.6
23F	40	10.6
19F	35	9.3
14	34	9.0
19A	21	5.6
23B	15	4.0
25F/38	14	3.7
18/(18A/18B/18C/18F)	11	2.9
34	8	2.1
12F/(12A/44/46)	8	2.1
15B/15C	8	2.1
13	7	1.9
1	6	1.6
10F/(10C/33C)	6	1.6
15B	6	1.6
12F	5	1.3
16F	4	1.1
38/25F/25A	4	1.1
3	3	0.8
4	3	0.8
11A/11D	3	0.8
9V/9A	3	0.8
10A	2	0.5
17F	2	0.5
août-39	2	0.5
15A/15F	1	0.3
15F	1	0.3
33F/(33A/37)	1	0.3
35F	1	0.3
7C/(7B/40)	1	0.3
7F/7A	1	0.3
PCR-NT	62	16.4
TOTAL	377	100.0

**Fig 2:**
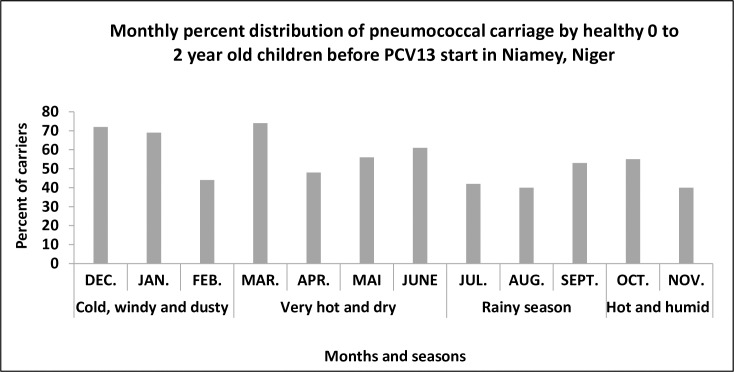
Bar plots showing percent distribution of pneumococcal carriage per month and per season among healthy, 0 to 2 year old children in Niamey, Niger.

The study was conducted across all seasons of the year and a temporal examination of data indicated significant variation in pneumococcal carriage with regard to seasons and months (*P = 0*.*001* and *P<0*.*001* respectively) **(**Fig 2). Carriage was additionally found significantly changing with age-groups (*P<0*.*001*), increasing from 44.8% (150/335) in children aged ≤3 months to 59.8% among those aged between 3 to 12 months (384/642) and decreased to 54.2% (117/216) in those aged ≥12 months. Significance of risk determinants associated with pneumococcal carriage was also analyzed first by univariate analysis and then by multiples regression models (Table 2). It was found that, age > 3 months significantly increase the risk of carrying SPN (a*OR = 2*.*3*, *P = 0*.*015*. Additionally, presence in family of at least one child aged less than 6 years likely constituted a risk factor for toddlers to carry a pneumococcus (*aOR = 2*.*1*, *P<0*.*001*). On the contrary, SPN carriage was not significantly found associated with birth weight < 2500g (*P = 0*.*973*), history of infectious disease or treatment with an antibiotic within 3 months to 7 days before the inclusion date (*P = 0*.*309* and *P = 0*.*395*), attendance of a daycare center or family (*P = 0*.*733*) or smoking environment (P = 0.057). Similarly, no substantial difference in SPN carriage was detected between ethnic groups of mothers (*P = 0*.*128*) and no ethnic group showed association with carriage in univariate analysis (*P-values >0*.*05*). However, in multiple regression analysis (Table 2), the Kanouri ethnic group revealed an important negative association with SPN carriage (*OR = 0*.*04*, *P = 0*.*006*).

**Table 2:** Univariate and multivariable analysis of risk factors association with *Streptococcus pneumoniae* carriage in 0 to 2 year-old children before PCV13 start in Niamey, Niger.

Factors			Univariate			Multivariate		
	No of subjects	SPN Carriers (%)	OR	P-value	95% CI	aOR	P-value	95% CI
Age > 3 months	858	501 (58.4)	1.7	**<0.001**	1.3–2.2	2.3	**<0.001**	1.4–2.9
Breast-feeding	1182	646 (54.7)	1.5	0.476	0.6–3.8	1.5	0.172	0.8–2.8
Presence of at least 1 Sibling aged < 6 years	673	410 (60.9)	1.8	**<0.001**	1.4–2.3	2.1	**<0.001**	1.5–2.9
Smoking environment	469	271 (57.8)	1.3	0.057	1–1.6	1.2	0.203	0.9–1.7
History of antibiotic treatment 3 months to7 days before enrolment	155	80 (51.6)	0.9	0.427	0.6–1.2	0.9	0.649	0.5–1.5
History of infection within the last 3 months	562	297 (52.8)	0.9	0.322	0.7–1.1	0.9	0.577	0.6–1.4
Attendance of daycare center or family	126	67 (53.2)	0.9	0.777	0.6–1.4	1.1	0.665	0.7–1.8
Being Male sex	629	339 (53.9)	1.0	0.685	0.8–1.2	0.8	0.684	0.6–1.1
Being premature	97	51 (52.6)	1.0	0.914	0.6–1.5	1.0	0.928	0.6–1.7
Ethnic group Zarma-Sonrai	589	337 (57.2)	1.2	0.071	1–1.6	0.4	0.255	0–1.6
Ethnic group Hausa	310	155 (50)	0.8	0.063	0.6–1	0.3	0.166	0–1.4
Ethnic group Kanouri	18	6 (31.6)	0.4	0.060	0.1–1	0.04	**0.001**	0–0.3
Burkinabe	36	25 (69.4)	1.9	0.882	0.9–4	1.2	0.807	0.3–5.4
Gurmantche	16	9 (56.2)	1.1	0.900	0.4–2.9	0.2	0.175	0–1.8
Fulan	34	17 (50)	0.8	0.604	0.4–1.6	0.8	0.850	0.1–4.8
Togolese	26	13 (50)	0.8	0.693	0.4–1.8	0.6	0.527	0.1–2.9
Tuareg	95	48 (50.5)	0.8	0.452	0.5–1.3	1.0	0.974	0.2–4.1
Beninoise	25	16 (64)	1.5	0.419	0.7–3	2.1	0.354	0.5–10.3

Legend: OR = Odds ratio; aOR = adjusted Odds ratio; CI = Confidence Interval. Bold figures represent significant factors

### Antibiotic Sensitivity

**Fig 3:**
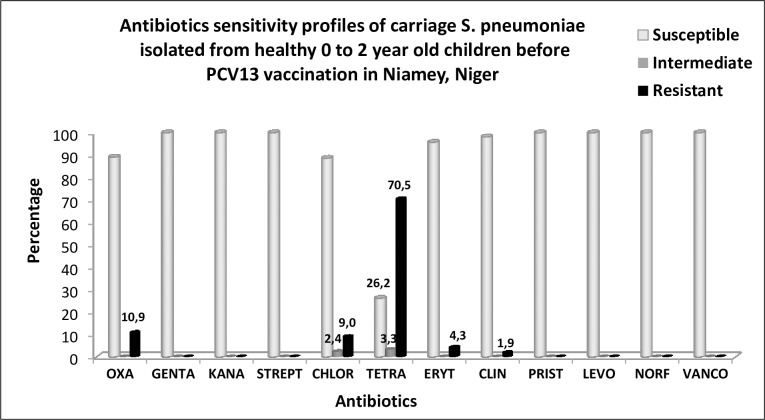
Antibiotic sensitivity profile of pneumococcal carriage isolates detected from healthy, 0 to 2 year old children before PCV13 start in Niamey, Niger. Legend: OXA: oxacillin, GENTA: gentamycin, KANA: kanamycin, STREPT: streptomycin, CHL: chloramphenicol, ERY: erythromycin, TET: tetracycline, CLIN: clindamycin, PRIST: pristinamycin, LEVO: levofloxacin, NORF: norfloxacin, VAN: vancomycin.

A total of 211/377 (56%) isolates were tested for their antimicrobial sensitivity. We detected 23/211 (10.9%) isolates with reduced sensitivity to penicillin (PRSP) out of which, 3 were fully resistant to penicillin. These PRSP isolates were mainly of serogroups/serotypes 19A (6/23), 6/(6A/6B/6D/6C) (4/23), 23A (3/23), 23F (2/23), 19F(2/23), 1 for each of serotypes 1, 14, 15B/15C and 12F(12A/44/46) and PCR-NT (3/23). The latter were additionally multi-resistant to chloramphenicol, erythromycin and tetracycline. Two and 12 other PRSP isolates also had an additional intermediate and full resistance to tetracycline respectively. Globally, resistance to chloramphenicol, erythromycin and tetracycline was respectively 11.4% (24/211), 4.3% (9/211) and 70.5% (148/210) (Fig 3). No resistance was however detected to gentamycin_**500μg**_ and to fluoroquinolones (ø Norfloxacin_**5μg**_ <7mm).

## Discussion

With GAVI assistance, pneumococcal conjugate vaccines (PCVs) have been successively introduced into immunization programs of many developing countries. These PCVs are expected to bring significant changes in the epidemiology of invasive pneumococcal diseases (IPD) and carriage in these settings. However, not all developing countries possess a solid surveillance system to monitor vaccine impact [13]. Carriage studies have been largely reviewed and found useful as a proxy method to monitor PCV impact, compared to expensive population-based surveillance [14, 15].

The present paper is the most recent report from Niger on study of pneumococcal carriage prior to PCV13 vaccine roll out. The data were collected from 2007 to 2008 and complement the study by Collard et al. [7] on invasive serotypes published in 2013 aiming to determine pneumococcal serotypes distribution and theoretically predict PCV13 vaccine coverage. For the determination of carriage serotypes and distribution in this study, we used the sequential multiples PCR algorithm designed by Collard et al [7]. The first challenge encountered was the inability of many frozen isolates to be re-grown, probably due to loss of viability as a result of repetitive electric power failure and subsequent temperature rise of deep freezers over time. The second challenge was the high rate of PCR non-typeable isolates (16.4%), which could be in large part those serotypes not covered by the adopted PCR design (Table 3). Additionally, the method was not able to discriminate some closely related serotypes. Globally, a rate of 54.5% (654/1200) carriage was detected, which is comparable to 50.6% rate observed in the same age group in Burkina Faso [16] and to 50% reported in Senegal [17]. Prevalence of SPN carriage in healthy children <5 years of age varied from 20% to 93.4% in developing countries and is generally higher than reported [9]. We analyzed various risk conditions for their eventual association with pneumococcal carriage using univariate and multivariable analysis. As generally reported by many studies, we found carriage significantly age dependent (*P<0*.*001*), increasing from 44.8% (150/335) in children aged ≤ 3 months to 59.8% among those aged between 3 to 12 months (384/642) and subsequently decreased to 54.2% (117/216) in those aged ≥12 months. But, statistically, children aged >3 months were found significantly (*P<0*.*001*) at risk of carrying SPN (a*OR = 2*.*3*). A possible reason among others to explain this is that in Niger, three months after delivery most mothers resume work activities or office and so need to entrust their babies to other adults or older children in the family for care. This increases contact with carriers and so the risk of babies to be contaminated. Age influence on carriage was previously reported in several studies carried out in Africa [1, 3, 18, 19]. Although pneumococcal carriage was not significantly found associated with none of the most important ethnic groups of mothers in univariate analysis, multivariable analysis showed children of Kanouri ethnic group mothers significantly (*P = 0*.*001*) less at risk of being colonized by a SPN (*aOR = 0*.*04*). We assume this is either due to unbalance sample size or to influence of other conditions (interaction). We recommend this phenomenon to be deeply examined in future studies in Niger with larger data on socio-demographic and traditional practices collected. With regard to changes in carriage due to seasons (Fig 2), a statistically significant difference was observed related to month (*P< 0*.*001*) and to season (*P = 0*.*001*) of the year. This could be explained by the fact that, Niger is in the heart of African meningitis belt where pneumococcal infections are endemic with SPN still being reported as second and most severe cause of meningitis after *Neisseria meningitidis* with similar pattern of seasonal variation. On the contrary, we did not find gender, history of infection or treatment with antibiotics and breastfeeding associated with carriage (*P-values >0*.*05*). Such observations were reported in studies from Sub-Saharan Africa, though, some authors found gender affecting pneumococcal carriage. Similarly, no significant association was detected between carriage and attendance of a daycare center or family. Because professional information was not collected in this study, we assume that mothers of the recruited children might be mostly housewives who have much time and stay longer at home caring for their babies, occasionally taking them to another family or daycare center only for a short time. Thus, the number of children attending daycare centers mainly recruited during routine vaccination in health center might be insufficient in size to show significant impact of daycare center attendance on SPN carriage.

**Table 3:** Serotypes not detectable by the Sequential Multiplex PCR adapted by Collars et al., 2013.

10B, 11(B, C), 13, 16A, 17(A), 19(B, C), 23A, 27, 28(A, F), 29, 31, 32(F,A), 33(B, C,D), 36, 41(A), 45.

Although not all serotypes are detectable by the adapted PCR design (Table 3), 32 different serogroups/serotypes were identified. The most prevalent ones were 6/(6A/6B/6C/6D), 23F, 19F, 14, 19A, 23B, 25C/38 and 18/(18A/18B/18C/18F) respectively representing 15.6%, 10.6%, 9.3%, 9%, 5.6%, 4%, 3.7% and 2.9%. PCR-NT isolates accounted for 16.4% (62/377) which is too high. We assumed that beside unencapsulated isolates, high proportion of serogroups/serotypes not amplifiable by the used PCR design might be in contribution. Nevertheless, with exception of PCR-NT isolates, the obtained results are comparable to those reported by Ba et al. [17] in Dakar, Senegal, by M.A Charvériat et al. in New Caledonia and with most studies conducted in under five year old children in low and middle-income countries [9,20]. In fact, since 1994, serotypes 6A, 6B, 23F, 19F and 14 have been reported as serotypes that colonize and infect children [21]. These serogroups/serotypes were previously detected by Collard et al. in the study of invasive pneumococci in Niger and reported them as the most prevalent cause of meningitis after serotype1 in < 2 year old children [7]. Although serotype1 was a major cause of meningitis in Niger [7], this study found 1.6% rate of carriage versus 4.3% rate detected by Collard et al. among similar age-group of children. The slight difference could be due to the fact that serotype1 SPN is seldom detected in healthy carrier individuals even in locations where it is the major cause of IPD [22]. Of the 32 serogroups/serotypes detected in this study, 11 were included in PCV13 and accounted for 57.3% (216/377 isolates) (Fig 1). This rate represented the theoretical PCV13 vaccine coverage predicted on carriage. It is low compared to 68.7% coverage rate reported by Collard et al. [7] and to 70% coverage generally reported by most studies in Africa [21]. The difference is again probably due to low detection of serotype1. The present study tested 211 carriage isolates for their susceptibility to antimicrobials and found 10.9% (23/211) rate of reduced sensitivity and 1.4% (3/211) rate of resistance to penicillin. These levels of pneumococcal resistance to penicillin were low, compared to 55.3% and 28% of respectively intermediate and full resistance to penicillin reported by Iliyasu et al., [23] in Northern Nigeria, a country sharing its largest border with Niger. Serogroups/serotypes found resistant to penicillin by this study were mainly 6/(6A/6B/6C), 19F and 23F. Clones of these serotypes have a worldwide history of multidrug resistance [24, 25]. Indeed, since the first report of penicillin-resistant SPN in Australia in 1967, and later in New Guinea (1974), South Africa (1977), and Spain (1979), a worldwide dissemination of these serotypes was observed with the geographical difference in prevalence. Most of these strains are frequently detected in carriage among infants and young children [16, 24]. In this study, we also obtained 3.3% and 70.5% of intermediate and full resistance to tetracycline, indicating circulation in carriage of tetracycline resistant isolates among children aged 0 to 2 year old. High level of resistance to tetracycline (84%) was already observed in clinical isolates of serotype1 SPN from Niger in which, *tet*(M) gene coding for resistance to tetracycline was found [26]. Though progressively being abandoned for its toxicity (National treatment guidelines) since 2012, we tested the sensitivity of isolates to chloramphenicol and found that 24/211 (11.4%) were fully resistant to this antibiotic. This resistance was found widely distributed to many serotypes among which were serotypes 6/(6A/6B/6C/ 6D), 19F, 15F and PCR-NT isolates.

## Conclusion

In this study, 54.5% (654/1200) rate of pneumococcal carriage was detected in healthy, 0 to 2 year old children prior to PCV13 vaccines start in Niger. In total, 377/654 isolates (57.6%) were typed allowing detection of 32 different serogroups/serotypes. Out of these, 11, cumulating to 57.3% (216/377) were PCV13 vaccine-types. Isolates with reduced sensitivity and resistance to penicicllin respectively represented 10.9% and 1.4% with some being multidrug-resistant and/or known to cause diseases in children. Age more than 3 months and living with siblings less than 6 years old were significant risk factors for pneumococcal carriage. These data are thus expected to help understand post vaccines start changes in the epidemiology of pneumococcal carriage and infections in children.

## Supporting Information

S1 File Study questionnaire form. (PDF)

S2 File Study Data. (CSV)
