# Supplementary material for: Serotype Distribution and Antimicrobial Sensitivity Profile of Streptococcus pneumoniae Carried in Healthy Toddlers before PCV13 Introduction in Niamey, Niger
Source: PLoS One. 2017 Jan 19;12(1):e0169547. doi: 10.1371/journal.pone.0169547 (PMC5245802; doi:10.1371/journal.pone.0169547)
Supplement: S1 File — (PDF) [file pone.0169547.s001.pdf]

ACIP Pneumocoques - Formulaire portage

Numéro d'inclusion: NIG-P-1
Type d'inclusion: Portage
Date d'inclusion: 05-juin-07

Lieu de recrutement: CSMI Yantala

Nom de l'enfant: SANDIWIDI
Prénom de l'enfant: BERNARD

Sexe: Masculin

Date de naissance: 21-nov-05 *ou* Age (en mois):

Poids de naissance:

Nombre d'autres enfants dans le foyer: 4
dont < 6 ans: 0

Origine ethnique déclarée
Ethnie 1: Mossi
Ethnie 2:

Antécédents infectieux dans les 3 derniers mois: Non
Maladie 1: / Préciser autre 1:
Maladie 2: / Préciser autre 2:
Maladie 3: / Préciser autre 3:

Antécédents d'antibiothérapie: Non
Antibiotique1: / Quelle ancienneté ?
Antibiotique2: / Quelle ancienneté ?
Antibiotique3: / Quelle ancienneté ?

Allaitement maternel: >= 2 mois
Garderie, crèche ou nourrice >4 h / semaine: Non
Environnement fumeur: Oui

Culture: S. pneumoniae

r: 1 sur 1200 Aucun filtre Rechercher
